# The Epigenetic Cytocrin Pathway to the Nucleus. Epigenetic Factors, Epigenetic Mediators, and Epigenetic Traits. A Biochemist Perspective

**DOI:** 10.3389/fgene.2017.00179

**Published:** 2017-11-27

**Authors:** Gemma Navarro, Nuria Franco, Eva Martínez-Pinilla, Rafael Franco

**Affiliations:** ^1^Department of Biochemistry and Physiology, Pharmacy School, Universitat de Barcelona, Barcelona, Spain; ^2^Centro de Investigación en Red, Enfermedades Neurodegenerativas, Instituto de Salud Carlos III, Madrid, Spain; ^3^Can Sunyolet, Martorell, Spain; ^4^Departamento de Morfología y Biología Celular, Facultad de Medicina, Instituto de Neurociencias del Principado de Asturias, Universidad de Oviedo, Asturias, Spain; ^5^Molecular Neurobiology Laboratory, Department of Biochemistry and Molecular Biomedicine, University of Barcelona, Barcelona, Spain

**Keywords:** nucleocrin, thermodynamics, state variables, inheritability, therapy, Alzheimer's disease, cancer, tumor therapy

## Abstract

A single word, Epigenetics, underlies one exciting subject in today's Science, with different sides and with interactions with philosophy. The apparent trivial description includes everything in between genotype and phenotype that occurs for a given unique DNA sequence/genome. This Perspective article first presents an historical overview and the reasons for the lack of consensus in the field, which derives from different interpretations of the diverse operative definitions of Epigenetics. In an attempt to reconcile the different views, we propose a novel concept, the “cytocrin system.” Secondly, the article questions the inheritability requirement and makes emphasis in the epigenetic mechanisms, known or to be discovered, that provide hope for combating human diseases. Hopes in cancer are at present in deep need of deciphering mechanisms to support *ad hoc* therapeutic approaches. Better perspectives are for diseases of the central nervous system, in particular to combat neurodegeneration and/or cognitive deficits in Alzheimer's disease. Neurons are post-mitotic cells and, therefore, epigenetic targets to prevent neurodegeneration should operate in non-dividing diseased cells. Accordingly, epigenetic-based human therapy may not need to count much on transmissible potential.

## Ancient origins

Discovery in the 1950s of the double helical structure of the DNA opened the era of everything being explained by Genetics/Molecular Biology, i.e., essentially in terms of the sequence of the genome. The lack of responses for the varied phenotypic (environmental, physiological, etc.,) aspects displayed by living beings has led to the fashionable “Epigenetics era.” For many scientists, the field is recent and epigenetic mechanisms consist of modifying the structure of histones and of DNA methylation patterns. However, the field has ancient and appealing origins and it contains more faces/cornerstones than usually considered. This Perspective article attempts to give a holistic view of factors and players in Epigenetics. The relevant goal to achieve epigenetic-based therapies probably awaits discovering essential clues and looking at them under an appropriate perspective.

Epigenetics' precursor word, “epigenesis,” was used to confront a current of thought (alive for many centuries), which assumed that different parts of the body of mammals were pre-formed in the spermatozoon (*preformation*). Both Aristotle and William Harvey who were born, respectively, 384 years before Christ and in 1578, share recognition for inventing “epigenesis.” The first putted a name (in Greek) to define the development from, for instance, a chicken egg to a chick with beak, legs, wings, etc., The second coined the English word for describing the same concept. A recent review shows the origins and evolution from epigenesis to epigenetics in a very attractive way (Deichmann, 2016). In the first part of the twentieth Century, the embryologist Conrad Waddington coined the term “Epigenetics” and, later on, funded the first “epigenetics laboratory.”

The term may be used as an adjective or as a noun. Riggs et al. (1996) as quoted in Haig (2004) defined epigenetics (noun) as: “*the study of mitotically and/or meiotically heritable changes in gene function that cannot be explained by changes in DNA sequence*.” A second definition was provided by Herring (1993) as quoted in Haig (2004): “*the entire series of interactions among cells and cell products which leads to morphogenesis and differentiation*.” Then, epigenetic may be used as an adjective of any non-genetic mechanism underlying morphogenesis and differentiation. The last sentence in the abstract of the inspirational Felsenfeld (2014) review states: “*Recent discoveries about the role of these mechanisms in early development may make it desirable to return to the original definition of epigenetics*.” As mentioned in Deichmann (2016), Waddington defined Epigenetics as “*the whole complex of developmental processes that lie between genotype and phenotype*.” We think that this definition applies to both tissue-specific phenotypes under development and for mechanisms providing phenotypic diversity in adults, for instance, among genotype-identical twins that are subject to different environmental, life-styles, etc., constraints.

Surely, it is worth reading seminal papers and books on this matter (Waddington, 1939, 1942, 1956; Haig, 2004; Felsenfeld, 2014) some of which are quite old (i.e., pioneering) and for this reason not included in the PubMed database. In particular, one article with a suggestive title “*The (dual) origin of epigenetics*” (Haig, 2004) guides the reader on how to start confronting the diverse epigenetic faces. Recommendable is also to take into account the sections devoted to Epigenetics in the book on the “four dimensions” of Evolution by Jablonka and Lamb (2002).

## Epigenetic factors

Britannica encyclopedia's definition of epigenetics “*the study of the chemical modification of specific genes or gene-associated proteins of an organism*” beautifully summarizes today's more fashionable epigenetic traits, namely DNA methylation and histone acetylation. The first one is a chemical modification that regulates gene expression i.e., that contributes to turn genes on or off. Analogously, post-translational modification (acetylation but also methylation) of histones, which are proteins that directly interact with DNA, is a chemical mechanism with significant impact on gene regulation. These and other similar -though less studied- mechanisms operate in the nucleus, close to DNA. Quite unlikely for histone acetylation marks, DNA methylation patterns may be clonally inherited, i.e., they may pass to daughter cells, meaning that epigenetic factors in a liver cell are transmitted to another liver cell, or the other way around: upon mitosis of a kidney/muscle/etc. cell, some DNA methylation traits are maintained in the two arising cells, i.e., they are clonally inherited. Recent epigenetic markers impacting on gene expression regulation are exosomes and atypical RNAs: micro RNAs (miRNAs) and long non-coding RNAs. At present, it is not known whether these factors may be clonally inherited but, importantly, exosomes are acting as endocrine factors. miRNAs are described in mammalian body fluids and, by definition, they are also endocrine factors. By the same token, can classical endocrine factors, which do regulate gene expression in target cells, be considered epigenetic factors?

## The poor knowledge on mechanisms

Today's fashionable epigenetic traits and the so-called epigenome are descriptors. A serious limitation to understand the real relevance of specific epigenetic traits is the lack of relevant information on mechanisms. For instance, why there are so many histone deacetylase genes and why, how and by whom DNA methyltransferases/demethylases and histone acetyltransferases/deacetylases become activated and deactivated. Also, the underlying mechanisms of the novel epigenetic mediators are still very obscure (Wiklund et al., 2010; Celluzzi and Masotti, 2016; Lange et al., 2017). We consider relevant that miRNAs and exosomes may act under endocrine paradigms. In fact, they are produced in a given organ/cell and reach another one by traveling via body fluids. In the case of exosomes, the gene regulation in the target cell(s) may be achieved by means of its RNA but also by other mediators that are contained in these vesicles. Accordingly, we find no reason to consider that hormonal regulation does not have epigenetic character, especially if we take into account that the endocrine regulation by which hormones act via receptors to affect gene expression is one of the best-known mechanisms of gene expression regulation.

## The cytocrin system. epigenetics within a whole-body framework

Hormonal regulation in mammals is categorized as endocrine, paracrine and/or autocrine. Regulation of cell phenotype in unicellular organisms by extracellular factors (hormones or nutrients) can only be autocrine, but the word is misleading since some of the regulators are produced by the cell while others are provided by the medium/environment. Actually, there is a need to denote the single-cell equivalent to endocrine system. Whereas, the suffix “crine” denotes something that is released to act from the outside, the cell machinery integrates and conveys all kind of extracellular signals to the cytoplasm and to the nucleus to regulate gene expression. We consider that the suffix “crin,” which refers to something that goes down as the crin of an animal, may be appropriate to describe this phenomenon in a general way. The underlying idea is to consider “crin” anything that goes from membranes inwards and can be applied to everything confined within a membrane (e.g., cell, nucleus, or mitochondria). Accordingly, the “cytocrin system” would describe the top-down effect of the different molecules impacting a unicellular organism or a single cell. Similarly, “nucleocrin” would describe the effect of the factors conveying cytoplasmic signals reaching the nucleus to be engaged in controlling gene expression. As mitochondria also has DNA, the equivalent word might be “mitochrin.”

As indicated above, nutrients are key regulators despite not being produced by cells. Accordingly, mechanisms that regulate cell life in a general way (nutrients constitute again a convenient example) reach DNA and histones first in a cytocrin and later in a nucleocrin fashion. Following the reasoning and considering that each single cell in a mammal has its own internal machineries, “nucleocrin” could be used to describe mechanisms that end up activating/deactivating factors directly affecting the chromosomal structure and function. Note the difference between “nucleocrin” and “nucleocrine,” which was a term coined by Radulescu (1995) to denote, in a cancer research context, how extracellular regulators may promote cell growth by interacting with nuclear proteins (tumor suppressors) (Radulescu, 1995, 2015). Moreover, we feel necessary to establish the mechanisms involved in transmitting extranuclear signals to the nucleus: directly (acting on transcription factors), and/or indirectly (acting on enzymes involved in managing epigenetic traits). We also consider more relevant to decipher the epigenetic mechanisms (triggers, activation/deactivation patterns, etc.,) than empirically describe methylation patterns, or that a given miRNA is increased in a given physiological circumstance or in a given disease (see below).

## From epigenetics in unicellular organisms to epigenetics in mammals

Anything impacting on a unicellular organism is prone to provoke a reaction i.e., a specific phenotype. One of such factors is the carbon source needed for survival. Depending on the growth medium, the phenotype of a given organism with a given DNA sequence could change. Taking a naïve approach, nutrients are non-genetic (i.e., they may be considered epigenetic) factors. Indeed, nutrients may modify DNA methylation patterns or histone acetylation as they lead to expression of specific transcription factor(s) and trigger gene regulation programs. The effect of some nutrients on gene expression is mainly known after the seminal identification of the lactose operon in bacteria by Jacob and Monod (1961).

To our knowledge, the field of *mammalian* epigenetics tends to forget the overall phenotype of an individual, which results from a given genome but multiple epigenetic overlapping factors and mechanisms acting under short (even in hours) and long-term (homeostatic/permanent-like) paradigms and during development but also in the adult individual. Epigenetics seems to be restricted to the development of multicellular organisms, i.e., to explain how a body containing billions of cells and dozens of cell types (with the same DNA) arises from a single cell. We propose to forget this limitation and expand “epigenetic” to any factor that, without involvement of DNA alterations, is affecting the phenotype of a mammal (or any of its cell types). Daughter cells resulting from a single ovum (having the same DNA) start to be different very soon, just when molecules, mainly nutrients, impact cells unevenly. Surely, one of the first mechanisms of control of gene expression in a developing embryo arises from concentration gradients, for instance, when oxygen reaches more concentration in one cell than in another. Oxygen gradients are essential for development. Why oxygen may be an epigenetic factor during development but not in adult life? Similar to oxygen, and as indicated above for unicellular organisms, nutrients coming from placenta and also metabolites (ATP, adenosine, amino acids, etc.,) that appear in the extracellular space are essential for development. Cell surface receptors are mediators that trigger, in cells having the same genome but becoming to *differentiate* (i.e., to be *different*), varied gene transcription programs that ultimately depend on the concentration of the endogenous agonist. This non-genetic, i.e., epigenetic, mechanism may rely on differential DNA methylation patterns but differential gene regulation is probably a more likely mechanism. It is true that the interest to link cell surface receptor activation to epigenetic traits has been quite low. On the one hand, to expand epigenetic options may lead to a more complex scenario, but we feel the contrary, i.e., that it will serve to clarification and consensus. On the other hand, either nutrient-based and hormone-mediated gene regulation are considered epigenetic mechanisms, or solid reasons are needed to exclude these classical mechanisms while accepting those of atypical RNAs or exosomes.

## Biochemistry-based proposal for more epigenetic factors

The lack of consensus on a clear-cut operative definition on Epigenetics impacts on the whole field. In their excellent essay Deans and Maggert (2015) emphasize that the term has “*multiple meanings, describing vastly different phenomena*.” We consider that Epigenetics may be as wide-ranged as possible to, subsequently, look for new names to describe some specific factors/mediators/mechanisms. Our proposal to expand the epigenetic window necessarily leads to more epigenetic players (in mammals). Taking disease risk as an example, prediction do require to know: (a) the DNA sequence, (b) factors directly affecting DNA/chromosomal structure and (c) factors indirectly affecting the expression of phenotype(s). Until now only factors in (b) are considered epigenetic; we think that factors in (c) are also epigenetic. Epigenetic is, in our opinion, the adjective for genetic-independent variables that shape our phenotype and even disease risk, disease progression and therapeutic management.

From a biochemist perspective, the Chemical aspects of any Biological issue should be taken into account. First of all, chemical reactions, from those in test tube to those underlying epigenetic mechanisms in living animals, are ruled by Thermodynamic laws and depend on “state” variables. As usual, temperature and pressure are the first to consider. The gene expression pattern and the resulting phenotype for a given genome depend on whether the living being lives at sea level or in a high mountain, and at below 0° in Siberia or at 35° in the tropic. Water, which was highlighted by Aristotle as crucial for life, is another factor but mainly in terms of humidity, for example, life in a desert or in humid Amazonia.

A non-exhaustive enough list of epigenetic factors, mediators and traits is provided in Table 1. The list focusses in mammals and in factors that depend on the own characteristics of these animals. Briefly, anything impacting on the five senses (sight, smell, touch, taste, and hearing) is, to a greater or lesser degree, affecting gene expression and, consequently, the phenotype. Take for instance light exposure; surely, gene expression for a same individual is different if living in Sweden -with few daily hours in winter and many daily hours in summer- or in any country in the Earth equatorial region. Food and life styles, education and social interactions, are further factors that may help in identifying novel epigenetic players, mechanisms and traits. In addition, epigenetic features already accepted should be positioned within the bigger picture.

**Table 1:** Non-exhaustive list of Epigenetic factors, mediators and traits in mammals.

**Factors**		**Observations**	**Example**	**Inheritability^b^**	**Endocrine-like**	**Underlying mechanism**
	Temperature	Thermodynamic (state) variable^a^	Living in Gobi desert *vs*. living in Alaska	No	No	-
	Pressure	Thermodynamic (state) variable^a^	Living at sea level *vs*. living in high mountains	No	No	-
	Gravity	Physical factor^a^	Living on Earth surface *vs*. living in Space station	No	No	-
	Water	Humidity	Living in a desert *vs*. living in Amazonia	No	No	-
	Light	Circadian rhythms	Living in Sweden *vs*. living in Equator	No	Yes (partially)	Partly known
Mediators	Hormones/neuro-transmitters	Regulating gene expression	Steroids	No	Yes	Partly known
	Nutrients	Regulating gene expression	Lactose operon^c^	No	Yes	Partly known
	Covalent DNA modification	Affecting DNA structure	DNA methylases	No	No	Unknown
	Post-translational modification of histones	Affecting chromatin/chromosome structure	Histone deacetylases	No	No	Unknown
	Transcription factors	Regulating gene expression	-	No	No	Partly known
	Atypical RNAs	Regulating gene expression	miR-29 micro RNAs, long non-coding (ncRNAs)?	?	Yes	Unknown
	Prion proteins	Affecting cell fate	Scrapie prion protein	Yes	?	Partly known
Traits	Histone modifications	-	Histone acetylation	?	-	-
	DNA modifications	-	DNA methylation	Yes	-	-
	Other	To be discovered	To be discovered	-	-	-

*Those that are currently more fashionable are highlighted in blue*.

a *In the absence of other impacting factors, state variables (also gravity) affect biological processes in a way that does not depend on the steps but on the initial and final conditions*.

b *Clonal (not transgenerational) inheritability, i.e., from one parental to the two after-mitosis daughter cells. Inter- and transgenerational transmission are not considered in this article*.

c *Although not operating in mammals it was the first described mechanism of gene regulation in response to nutrient availability*.

## Inheritance and transgenerational transmission issues

Inheritance is at the center stage in Epigenetics. Waddington was interested in developmental mechanisms but did not care much on inheritability, i.e., it was not fundamental for his idea of Epigenetics. Holliday redefined Waddington's concepts and convinced many scientists that inheritance was a necessary aspect in Epigenetics (Holliday, 1984, 1990, 1993, 2005, 2006). In fact, the need of inheritable epigenetic characters is one of the problems in reaching consensus. On the one hand, epigenetic traits are first discovered and subsequently *forced* to be inheritable. On the other hand, it is doubtful that the novel epigenetic traits are inherited. The issue of inheritance is difficult to reconcile and, from a biochemist point of view, it should be secondary if not forgotten. Many of the differential traits found even in identical monozygotic twins must be considered as epigenetic and not necessarily acquired by inheritance mechanisms during development.

Despite DNA methylation patterns may be clonally inherited there is a reprograming of such patterns in the egg/embryo; they are erased except in few imprinted gene sequences (Seisenberger et al., 2012; Wasson et al., 2013; Monk, 2015; Zhou and Dean, 2015). Accordingly, histone modifications or DNA methylation are very unlikely transgenerational epigenetic mechanisms. In the absence of a breakthrough finding it will take time to confirm epigenetic transgenerational transmission and the underlying mechanisms. One of few examples in mammals derives from the study of phenotypic traits in humans conceived in the 1944–1945 Dutch famine (Veenendaal et al., 2013), in which health, weight, body mass index rate, etc., of individuals conceived from poorly feed parents or from well-feed parents were compared. One interesting finding was a higher adiposity in the offspring of prenatally undernourished fathers but not mothers (Veenendaal et al., 2013). The topic has provided reviews where potential mechanisms and myths on this topic are presented and discussed (Dias and Ressler, 2014; Heard and Martienssen, 2014). We raise the subject here because the candidates for transgenerational transmission are the novel epigenetic mediators: miRNAs and exosomes. They may travel from a given somatic cell to the ovum or the spermatozoon to achieve the transgenerational transmission. In this sense, these epigenetic mediators may be inherited once integrated in spermatozoon precursors but, very importantly, its epigenetic action is endocrine as they should reach the spermatozoon precursors from elsewhere in the human body. In the eventual case of maternal transmission, the inheritability of epigenetic traits is very unlikely as ovules do not divide but are present since early steps in mammalian life span, i.e., the epigenetic factors must reach ovules one by one from elsewhere in the female body.

## Epigenetic-based therapy: targeting non-inherited epigenetic traits?

Leaders in epigenetic research are expanding the sentence: “we are what we eat” to something like “we are what parents, grandparents, etc., ate.” In fact, Sales et al. (2017) compile evidence to sustain that epigenetic transgenerational transmission may be the basis for “*non-genetic molecular legacy of prior environmental exposures and influence transcriptional regulation, developmental trajectories, and adult disease risk in offspring*.” The possibility of transmissible epigenetic factors that may impact on the disease risk of offspring, even across generations, is presented in several reviews (e.g., Nadeau, 2009; Somer and Thummel, 2014; Hur et al., 2017; Weber-Stadlbauer, 2017). However, in daily practice clinicians assume that the inherited risk of disease is due to DNAs inherited from father and/or mother. This assumption seems appropriate for a variety of diseases, for instance a higher risk of breast cancer in daughters from mothers having had the disease would be likely due to genetic factors. In other cases, where the genetic link is not so evident, the epigenetic one should be considered. Expecting to increase our knowledge on mechanisms linking epigenetic transmission and disease risk, the research has devoted to target epigenetic mechanisms to cure/combat diseases in gaining momentum. Accordingly, one of the most sought potential of Epigenetics is to translate preclinical research into “epigenetic” medicines.

The most studied paradigmatic case in epigenetic-based therapy is cancer. Evidence of epigenetic trait alterations in cancers was expectable and, in fact, provided in many studies. There are high expectations on targeting epigenetic DNA methylation or histone acetylation to combat cancer and in a recent review Perri et al. (2017) describe how anti-cancer therapeutic approaches may take advantage of epigenetic control of cell expression. In practice and as superbly reviewed by Flavahan et al. (2017) fulfillment of expectations would require “…*test, validate (or refute) conceptual and mechanistic models for cancer epigenetics, and place them in context with prevailing genetic models*.” At present, epigenetic traits may help in better classification of cancer but the therapeutic prospects are poor.

The inheritability of epigenetic mechanisms should be put under a proper perspective; it appears as irrelevant in fighting cancer since anti-cancer therapy attempts to kill malignant cells before providing more daughter cancer cells. Epigenetic mechanisms cannot be inherited in cells that do not divide; neurons are the paradigm of such cells. Surely, there is now accepted that a certain degree of neural development exists in some restricted areas of the adult brain but, such possibility is very limited in the aged brain and very compromised in diseases of the central nervous system.

In our opinion, a fixation on cancer is limiting the advance in epigenetic-based therapies for other diseases, e.g., neurodegenerative, in which cells should survive instead of being killed. Epigenetic changes impacting on degenerating neurons do not have much chance to be transmitted to daughter cells. Current attempts to combat neurodegenerative diseases, mainly Alzheimer's, address a epigenetic mechanism (histone acetylation, see Cuadrado-Tejedor et al., 2013 for review) that is common to affected neurons and that is not necessarily transmitted from cell to cell. It is true that transcellular transmission of a pathogen triggering similar epigenetic responses in connected neurons cannot be ruled out. But even if this is the case, therapies based in epigenetic traits should not essentially assume that the targeted trait is inheritable/inherited or not. Other players may be identified in the future, that also will tell which ones are really operating in a physiologically relevant way and whether they may constitute targets to combat diseases.

## Author contributions

GN and EM-P have searched for the literature and contributed to the writing. NF has contributed to the order of sections, subsections and Table construction, i.e., to the overall design. RF conceived the idea of the Perspective, contributed to the writing and did the coordination.

### Conflict of interest statement

The authors declare that the research was conducted in the absence of any commercial or financial relationships that could be construed as a potential conflict of interest.
